# Editorial Notes

**Published:** 1927

**Authors:** 


### Editorial Notes.

**The President in Canada.**

Dr. A. L. Flemming, during his year of office as President of the Bristol Medico-Chirurgical Society, received the high compliment of an invitation from the Canadian Medical Association to deliver an address on Anæsthetics at the Annual Meeting of the Association, held in Toronto in June last. Bristol may well congratulate itself on the choice of Dr. Flemming to represent the Mother Country at this important meeting in our oldest Dominion. Dr. Flemming was greatly impressed by the cordial reception with which, as a delegate of the British Medical Association and representative of the University of Bristol, he was greeted in professional and social circles in Canada. He reports with much satisfaction that on several occasions he heard this Journal spoken of in appreciative terms.

**The Sheriff of Bristol.**

Major J. A. Arrowsmith-Brown, D.S.O., has accepted the office of Sheriff of Bristol this year, and it is with particular pleasure that the Journal Committee offers its congratulations to the head of the firm of J. W. Arrowsmith Ltd., our publishers. Major Arrowsmith-Brown has, like his uncle, the late Mr. J. W. Arrowsmith, displayed the keenest interest in the progress of the University, and as Secretary of the Colston Research Society he has worked unremittingly to promote scientific research in Bristol. His gallant record during the War and his zeal for the welfare of our city leave us in no doubt that he will occupy the ancient and royal office of Sheriff in such a manner as to enhance its dignity and win the approbation of his fellow-citizens.

**Opening of the Henry Herbert Wills Physics Laboratory.**

ON October 21st the Henry Herbert Wills Physics Laboratory at the University was declared open by Sir Ernest Rutherford, President of the Royal Society, in the presence of the Chancellor of the University, Lord Haldane, and many distinguished British and foreign guests. For the foundation of this building, which is situated on the Royal Fort Estate, the late Mr. H. H. Wills gave £200,000 to the University. The Laboratory, finely designed and richly equipped, forms a noble addition to the University. There is still another important building in progress, the men's hostel at Downside on Durdham Down, which is nearly completed. Few modern Universities have buildings that can compare with Bristol's, and as succeeding generations look upon the buildings they cannot fail to speak with gratitude of the munificence of the Wills family.

**Postgraduate Medical Work.**

DURING September the University Postgraduate Committee for Clinical Studies arranged a fortnight's intensive course at the Royal Infirmary and General Hospital.

The Regional Medical Officer conducted the negotiations between the Ministry of Health, the various Insurance Committees and the University.

Fifteen panel practitioners attended from the counties of Shropshire, Herefordshire, Worcestershire and Gloucestershire.

This is the second course of the kind held at Bristol. In 1926 the class was limited to Somerset.

**The Annual Service for the Medical Profession.**

THE Annual Service for the Medical profession was held on Sunday, October 16th, in Bristol Cathedral. A large number of the medical and nursing professions were present. The offertory was put at the disposal of the Diocesan Board of Finance. The preacher[1] was the Right Rev. Dr. J. H. B. Masterman, Bishop of Plymouth, who took for his text the words: "What is man, that Thou art mindful of him?" (Psalm viii. 4). "The special work of doctors," he said, "brought them into contact with two sides of human nature. Their first concern was with the physical nature of man, with that nature which linked them with the whole system of organic evolution." The Bishop referred to a recent restatement of the Darwinian hypothesis, and remarked that the echo of this old controversy aroused little interest to-day. We were not profoundly interested in the ancestry of man except in so far as it had a vital bearing on his destiny. But in their contact with human nature every doctor must become in some measure a psychologist, and though scientific psychology was only in its infancy, it had at least

[1] The Report of the Bishop's sermon is taken from the *Bristol Times and Mirror* of Monday, October 17th.

taught them that soul-stuff was not resolvable into terms of matter. It was sometimes said that men had lost something of the desire for continued existence which seemed to Plato the strongest argument for immortality. He thought it was true that the best men no longer regarded the permanence of human personality as in itself the highest good. Man's right to survive depended on the belief that every personality in which the moral values had developed represented an enrichment of the ultimate reality. It was the conservation of values that was the supreme concern of the spiritual universe. So we reached the threshold of Christian theology, for it was in the Incarnation that we found the final vindication of those moral values, and salvation began for every man when he began to make those values his own. It was surely true that the Christian doctrine of the Incarnation did give to the whole world-progress a meaning and a value that we could find nowhere else. Man's ancestry went back to the dust, but his goal was the glory of God.

